# Analysing the High Strain Rate Behaviour of Cortical Bone with the Image-Based Inertial Impact (IBII) Test

**DOI:** 10.1007/s40870-025-00478-6

**Published:** 2025-06-06

**Authors:** L. Fletcher, F. Pierron

**Affiliations:** 1 Faculty of Engineering and Physical Sciences, University of Southampton, Southampton, UK; 2 MatchID NV, Ghent, Belgium

**Keywords:** Bovine bone, High strain rate, Grid method, Digital Image Correlation, IBII test

## Abstract

Traumatic bone fractures can occur under high strain rate loading. However, there is minimal high strain rate data available for cortical bone. Most existing data for the high strain rate properties of cortical bone has been obtained using the split-Hopkinson bar (SHB) under compressive loading. The SHB method requires that the test sample is in a state of quasi-static equilibrium which is difficult to achieve for quasi-brittle material (e.g. bone) loaded in tension. Recently, the Image-Based Inertial Impact (IBII) test was developed specifically for analysing the elastic stiffness and tensile failure stress of materials under high strain rate loading. Therefore, the overall aim of this study was to apply the IBII test to obtain new high strain rate data for the orthotropic stiffness components and tensile failure stress of cortical bone. Bovine cortical bone samples were tested parallel (longitudinal) and perpendicular (transverse) to the long axis of the bone. Using this test data it was possible to obtain high strain rate data for all four in-plane stiffness components as well as tensile failure stress. The results of this study provided an average longitudinal stiffness of 26 *GPa* at an effective strain rate of $1150~s^{-1}$ and transverse stiffness of 15.2 *GPa* at an effective strain rate of $1300~s^{-1}$. Using paired quasi-static samples, this represents a $22\%$ rate sensitivity for the longitudinal samples and a $5.5\%$ rate sensitivity for the transverse samples. Slight misalignments of the projectile made it possible to obtain the shear modulus for some samples with an average shear modulus over all samples of 6.9 *GPa* with an effective strain rate of $1200~s^{-1}$. The average tensile failure stress of the longitudinal samples was 146 *MPa* and 53.6 *MPa* in the transverse direction at a strain rate of $5200~s^{-1}$.

## Introduction

Many cases of bone fracture occur under dynamic loads. The strain rate experienced during a traumatic fracture event can vary widely from that experienced during a fall through to the much higher strain rates applied by ballistic or blast loading. Having an accurate material model including rate dependence is key for understanding the cause of traumatic fracture events and developing new protective equipment [1]. Despite this, the majority of mechanical test data for bone is available at quasi-static strain rates [2, 3] and most of the data available at higher strain rates focuses on compressive loading [4, 5].

Bone has a hierarchical structure which can be considered as a three phase composite comprised of collagen, calcium hydroxyapatite and water [2]. This unique structure leads to complex anisotropic material behaviour. At the macro-scale bone can be classified into two distinct types, the foam like trabecular bone and the solid cortical bone. Here we focus on cortical bone as it can be treated as a homogeneous continuum at the macro-scale whereas the separation between material and structure is much less clear for trabecular bone. More advanced analysis techniques are needed to separate the material response of trabecular bone from the overall structural response. Therefore, accurately obtaining the high strain rate response of trabecular bone is still an open research problem. As the present work analyses the high strain rate response of cortical bone the remaining discussion is focused on this type of bone.

Given that bone has a polymeric phase in the form of collagen as well as a fluid phase it is expected that the material properties vary with strain rate [4, 5]. However, there is considerable uncertainty about the magnitude of the rate sensitivity with most data being limited to compressive loading. A summary of existing high strain rate data for bone is given in Ref. [5]. Experimental data for cortical bone under high strain rate loading is generally obtained using the split-Hopkinson bar (SHB) technique [5–11]. In order to obtain accurate data with this method it is necessary to assume the sample is in a state of quasi-static equilibrium so that inertial effects can be neglected [12]. Further, inertial effects are most prominent in the early portion of an SHB test making it difficult to accurately measure the elastic response at strain rates higher than a few hundred $s^{-1}$. Additional difficulties with the SHB method come from the need for accurate sample strain measurement. Previous studies have shown that traditional SHB theory can lead to highly inaccurate strain values when on-sample strain measurement is not used [13–15].

The work presented in [5] addressed some of the issues related to SHB testing of bone as the authors used direct strain measurement on the sample with digital image correlation (DIC). Overall, the results of this study showed an increase in elastic modulus and compressive failure stress with strain rate. For the elastic modulus the rate sensitivity was minimal for the longitudinal modulus but more pronounced for the transverse modulus. However, both the longitudinal and transverse compressive failure stresses increased significantly with strain rate. The experiments in [5] focused on the compressive response of cortical bone under high strain rate loading with small cuboid samples. However, compressive failure in quasi-brittle materials like cortical bone always takes place because of an instability and therefore, are difficult to exploit in terms of intrinsic material property. Practical considerations involved in gripping the sample make testing samples with the SHB in tension much more difficult. Furthermore, the increased sample size required for the tensile SHB method makes it more difficult to satisfy the assumption of quasi-static equilibrium and for quasi-brittle materials (e.g. bone) the sample can fail before inertial effects have damped out. Therefore, there is a clear need for an alternative test method for analysing the high strain rate tensile failure stress of bone.

The most recent generation of high strain rate test methods has focused on leveraging the advances in ultra-high speed imaging technology (frame rates of 1 *MHz* or more). These new methods use full-field measurements combined with an inverse identification technique to obtain material properties at high strain rates. One such class of new methods are the so-called ‘inertial’ techniques which use acceleration fields to provide force information without the need to assume quasi-static equilibrium. These inertial methods have been successfully used to investigate the high strain rate behaviour of a wide variety of materials including: concrete/rock [16–18], cermets [19], carbon fibre composites [20–22], polymers [23, 24], metals [25], polymer foams [26, 27] and rubbers [28–30].

The aim of this work is to use the latest generation of image-based high strain rate test methods to obtain new data for the dynamic response of cortical bone with a focus on the rate sensitivity of all stiffness components and obtaining the tensile failure stress. This is also the first time the IBII test has been applied to an orthotropic material where both elastic and shear moduli are expected to exhibit rate dependence. This paper begins with a brief overview of the virtual fields method (VFM) theory. Next, the experimental methodology and data processing procedure for the IBII test are described. The final sections of the article discuss future work and limitations before summarising the main conclusions.

## The Virtual Fields Method

The virtual fields method (VFM) is a technique for extracting the constitutive response of a material using full-field measurements (e.g. DIC or the grid method). The VFM uses the principle of virtual work, given by:

1 $$\begin{aligned} \overbrace{\int _{\partial V} \! \varvec{T} \cdot \varvec{u^*}\,\textrm{d} S + \int _{V} \! \varvec{b}\cdot \varvec{u^*}\,\textrm{d} V }^{W_{ext}^*}- \overbrace{\int _{V} \! \varvec{\sigma } : \varvec{\varepsilon }^*\,\textrm{d} V}^{W_{int}^*} = \overbrace{\int _{V} \! \rho ~\varvec{a}\cdot \varvec{u^*}\,\textrm{d} V}^{W_{acc}^*} \end{aligned}$$

where $\varvec{T}$ is the traction vector and $\varvec{u^*}$ is the virtual displacement vector from which the virtual strain tensor is derived using: $\varepsilon _{ij}^* = \frac{1}{2} \left( \frac{\partial u_i^*}{\partial j} + \frac{\partial u_j^*}{\partial i}\right) ~,~i,j = x,y$. The external surface area of the object of interest is denoted $\partial V$ and its volume *V*. The body force vector is given by $\varvec{b}$, the Cauchy stress tensor by $\varvec{\sigma }$ and the acceleration vector is $\varvec{a}$. Finally, the material density is denoted $\rho$.

**Fig. 1:**
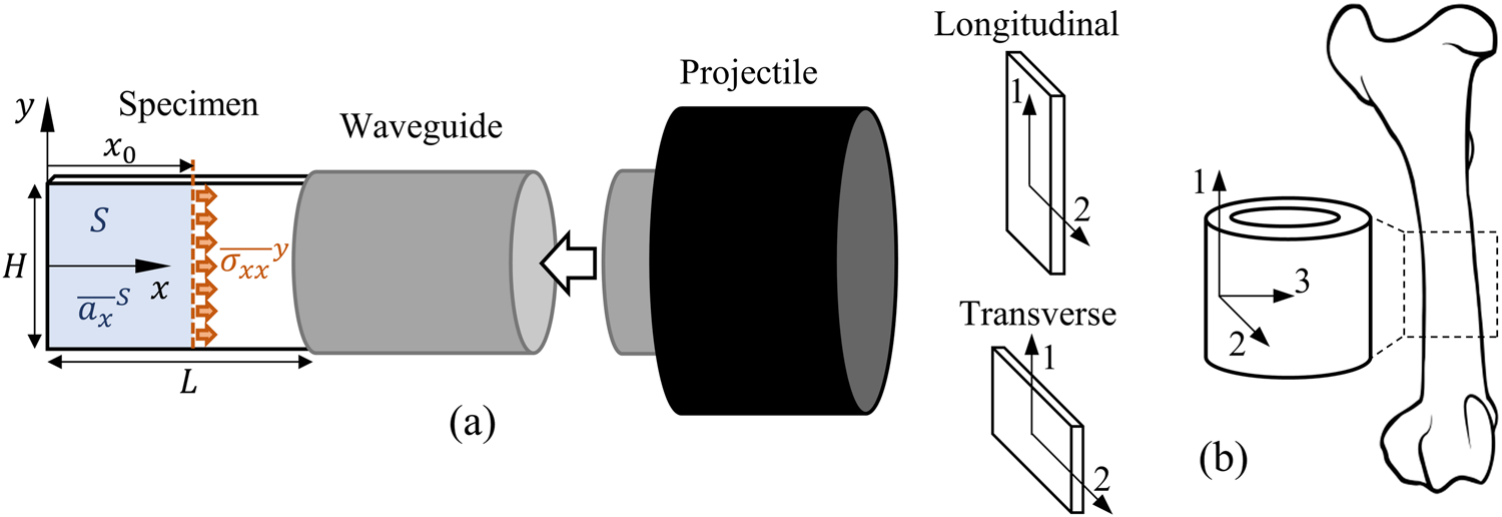
**a** Schematic of the IBII test configuration and **b** orientation of the bone samples with respect to the cortical shaft

For the typical IBII test configuration shown in Fig. 1a we have a thin plate-like sample which is loaded in-plane with full-field measurements providing kinematic information on the surface of the sample. Therefore, a number of assumptions are made to reduce the principle of virtual work to two dimensions. This includes: (1) the sample is in a state of plane stress, (2) the kinematics are uniform through the sample thickness, (3) the sample thickness is constant and (4) the density does not vary in space or time (i.e. the density is homogeneous and no shocks are present). For compactable material, it is however possible to use the strain measurements to update the density as done in [27]. For the IBII configuration body forces only come from gravity which is negligible here so Eq. 1 reduces to:

2 $$\begin{aligned} \int _{l} \varvec{T} \cdot \varvec{u}^* \textrm{d}l - \int _S \varvec{\sigma } : \varvec{\varepsilon }^* \textrm{d} S = \rho \int _S \, \varvec{a} \cdot \varvec{u}^* \textrm{d}S \end{aligned}$$

where the external virtual work is a evaluated over the perimeter of the test sample *l*, whereas the internal and acceleration virtual work are evaluated over the surface of sample *S* where full-field measurements are available.

In order to use Eq. 2 for material property identification, two further pieces of information are required. The first is a constitutive law and the second is a set of virtual fields. Here we consider the case of linear elastic orthotropy so the use of Eq. 2 with appropriate virtual fields produces a set of linear equations which are easily solved for the material stiffness parameters. The following sections outline the linear elastic orthotropic constitutive law as well as different approaches for using virtual fields for stiffness and failure stress identification.

### Constitutive Law

The elastic response of bone is normally considered to be transversely isotropic with the plane of isotropy being normal to the long axis of the bone (i.e. the radial-circumferential plane). Here we adopt the material axis convention given in Fig. 1b where the superscript ‘1’ refers to the axis aligned with the long axis of the bone and ‘2’ is transverse to the long axis (in this case aligned with the circumferential direction). For the IBII test, thin plate-like samples are considered along with the assumption of plane stress. Therefore, a plane stress linear elastic orthotropic constitutive law is used, as follows:

3 $$\begin{aligned} \begin{bmatrix} \sigma _{xx} \\ \sigma _{yy} \\ \sigma _{xy} \\ \end{bmatrix} = \begin{bmatrix} Q_{11} & Q_{12} & 0 \\ Q_{12} & Q_{22} & 0 \\ 0 & 0 & Q_{66} \\ \end{bmatrix} \begin{bmatrix} \epsilon _{xx} \\ \epsilon _{yy} \\ \gamma _{xy} \\ \end{bmatrix} \end{aligned}$$

where $\sigma _{ij}$ are the components of the stress tensor, $\epsilon _{ij}$ are the components of the small strain tensor and $\gamma _{xy}$ is the engineering shear strain which is twice the tensorial shear strain: $\gamma _{xy}=2\epsilon _{xy}$. The stiffness components are denoted $Q_{mn}$ and are related to the elastic/shear moduli and Poisson’s ratios as follows:

4a $$\begin{aligned} Q_{11}&= \frac{E_{11}}{1-\nu _{12} \nu _{21}} \end{aligned}$$

4b $$\begin{aligned} Q_{22}&= \frac{E_{22}}{1-\nu _{12} \nu _{21}} \end{aligned}$$

4c $$\begin{aligned} Q_{12}&= \nu _{21} Q_{11} = \nu _{12} Q_{22} \end{aligned}$$

4d $$\begin{aligned} Q_{66}&= G_{12} \end{aligned}$$

where $E_{11}$ is the longitudinal elastic modulus and $E_{22}$ is the transverse elastic modulus. The major Poisson’s ratio is given by $\nu _{12}$ and the minor Poisson’s ratio by $\nu _{21}$. Finally, the shear modulus is given by $G_{12}$. Given the co-ordinate system shown in Fig. 1b, the constitutive law described above holds for the case of a bone sample impacted in the direction of the longitudinal axis. For the case of a sample impacted in the transverse direction, the $Q_{11}$ and $Q_{22}$ terms in Eq. 3 are simply interchanged. Having selected a constitutive law, the next step in using the VFM for material property identification is the selection of virtual fields as discussed in the following section.

### Virtual Fields for Stiffness Identification

In this work, two types of virtual fields are used. The first are simple rigid body virtual fields that relate averages of the acceleration fields to the average stress as described in [19–21]. The second type of virtual fields are the special optimised virtual fields described in [31, 32] which are formulated based on a piecewise finite-element-like mesh. Here, we outline the theory for the simple rigid body virtual fields to illustrate the concepts.

The first rigid body virtual fields considered here describes a simple rigid translation along the ‘x’ axis: $u_x^*=1~,~u_y^*=0$ with null virtual strains. Substituting this virtual field into Eq. 2 gives:

5 $$\begin{aligned} \int _{l} \sigma _{xx} \textrm{d}l = \rho \int _S \, a_{x} \textrm{d}S. \end{aligned}$$

When full-field measurement are used the integrals in Eq. 5 can be approximated using the rectangle method as follows:

6 $$\begin{aligned} \overline{\sigma _{xx}}^{y} = \rho x_0 \overline{a_x}^{S} \end{aligned}$$

where $\overline{\sigma _{xx}}^{y}$ is the spatial average of the axial stress over the cross section located at position $x_0$ from the free edge and $\overline{a_x}^{S}$ is the surface average of the acceleration from the free edge to the axial slice at $x_0$. Hereafter, the overline notation coupled with superscript *y* indicates a spatial average over an axial cross section and the overline notation coupled with superscript *S* indicates the spatial average over a surface. This equation has been termed the ‘stress-gauge’ equation as it allows for the calculation of the average stress from the acceleration field with the density of the material acting as a load-cell calibration factor.

The stress-gauge equation can be used for stiffness identification when it is coupled with a constitutive law. Consider the ‘11’ component of Eq. 3 for a longitudinal sample: $\sigma _{xx} = Q_{11}(\epsilon _{xx} +\nu _{21} \epsilon _{yy})$. Therefore, when the material response is linear elastic, plotting the average stress $\overline{\sigma _{xx}}^y$ against the average strain $\overline{\epsilon _{xx} +\nu _{21} \epsilon _{yy}}^y$ produces a straight line with a slope equal to $Q_{11}$. An obvious drawback of this approach is that it requires that the minor Poisson’s ratio is known, or that the stress state is purely uniaxial, leading to the identification of $E_{11}$ instead of $Q_{11}$. One way of avoiding this is to use the generalized stress–strain curve approach as outlined in [33]. Unfortunately, for the simple aligned impact case considered here, the response is not heterogeneous enough to activate all the terms required to calculate the generalized stress–strain curves. In the future it should be possible to design a more interesting test configuration (e.g. using geometrical features or impacting over half the sample height) to intentionally create richer data for stiffness identification but for now the focus is on stiffness and tensile failure stress identification for aligned samples. Thus, another approach is required to extract the Poisson’s ratio.

An alternative approach for stiffness identification for IBII tests is the use of special optimised virtual fields. This is an automated procedure which produces unique virtual fields for each material parameter of interest that are tailored to minimise the effects of strain noise. A detailed description of the theory behind these virtual fields is beyond the scope of this paper so the interested reader is referred to [31, 32]. Here the special optimised virtual fields are formulated based on a piecewise mesh with virtual boundary conditions set to enforce $\varvec{u^*} = 0$ at the impact edge. These virtual boundary conditions are chosen to remove the contribution of the unknown impact force cancelling out the external virtual work term (recall Eq. 2) and leaving an equation which relates the internal virtual work to the acceleration virtual work through the unknown stiffness components.

Recently, it was shown that slight projectile misalignments in IBII experiments can generate a sufficient shear response to allow for the identification of the shear modulus using a vertical translation rigid body virtual field [34]: $u_x^*=0~,~u_y^*=1$ with null virtual strains. Following the same reasoning as for Eq. 6, the shear stress-gauge equation is obtained:

7 $$\begin{aligned} \overline{\sigma _{xy}}^{y} = \rho x_0 \overline{a_y}^{S} \end{aligned}$$

where $\overline{\sigma _{xy}}^{y}$ is the average shear stress at the cross section of interest at $x_0$ and $\overline{a_y}^{S}$ is the surface average of the acceleration. For the linear elastic orthotropic constitutive law given in Eq. 2.1, the shear stress response is only dependent on the shear strain. Therefore, the shear modulus, $G_{12}$, can be obtained by linearly fitting the plot of average shear stress $\overline{\sigma _{xy}}^y$ against average shear strain $\overline{\gamma _{xy}}^y$.

### Virtual Fields for Strength Identification

In the case of linear elastic behaviour, even if the stress/strain states are not uniform through the width, the stress gauge equation will provide the right slope. In other words, one can add together mechanical states that are linear even though each point along the width may be at a different location on the stress–strain line. However, to obtain the failure stress, the ‘stress-gauge’ equation needs to be used to approximate the local stress where failure initiates. If the stress distribution is not uniform through the width, then the stress average through the section will not correctly represent the local stress where failure occurs. It is possible however to enrich this description by adding an extra rigid body virtual field representing an in-plane rigid body rotation. Combining together the equations obtained with the three rigid body virtual fields, a linear approximation of the axial stress is obtained, as described in [19, 21, 22]. The resulting equation is termed the ‘linear stress-gauge’ (LSG):

8 $$\begin{aligned} \sigma _{xx}(LSG) = \rho x_0 \overline{a_{x}}^{S} + \frac{12 \rho x_0 y}{H^{2}} (\overline{a_{x} y}^{S} - \overline{a_{y} x}^{S} + x_0 \overline{a_{y}}^{S}) \end{aligned}$$

where the first term is equivalent to the stress-gauge equation derived previously (see Eq. 6). The second term describes the slope of the axial stress distribution over the specimen height in terms of weighted surface averages of the acceleration. This equation gives a richer estimate of the $\sigma _{xx}$ stress along the width and can be used for local failure stress identification.

Given that Eq. 8 is only a linear approximation of the axial stress the obvious question is ‘How accurate is the linear approximation compared to the true underlying stress field?’. Fortunately, all components of the strain tensor are available when using full-field measurements so this can be checked using the strains coupled with the previously identified stiffness components. When the material response is linear elastic the axial stress calculated from the strains and stiffness components should closely match that obtained with the linear stress-gauge equation. Therefore, an evaluation of accuracy of the linear stress-gauge equation (Eq. 8) is obtained by comparing it to the $\sigma _{xx}$ stress calculated from the strains in compression and unloading up to the point of failure (i.e. when the response is linear elastic).

The failure stress identification process involves several steps. The first step is to identify the location of the first fracture using the time sequence of the raw strain maps. A virtual stress gauge is then constructed and the temporal evolution of the average stress taken from the linear stress-gauge equation is compared to the average stress calculated from the strains for the local region where the first crack forms. The tensile failure stress is then taken as the peak tensile stress averaged over the virtual stress gauge. This approach has been previously used to obtain a local estimate of the tensile failure stress of brittle and quasi-brittle materials such as carbon fibre composites [21, 22] and tungsten carbide [19].

## Experimental Method

### Bone Samples

Three bovine femurs and one bovine tibia were obtained from a local meat wholesaler, from four different animals that will be referred to as C1–C4. Immediately upon obtaining the bones, the middle of the cortical shaft was sectioned into two pieces using a bandsaw. The upper and lower parts of the cortical shaft where then sectioned into quadrants using the band saw. Each quadrant was then wet-machined using a low speed diamond saw to rectangular plates with nominal geometry of $20 \times 12 \times 2~mm$. At all stages of the sample preparation process, the samples were stored in gauze soaked in Phosphate Buffered Saline (PBS) at $-20\,^{\circ }$C.

**Fig. 2:**
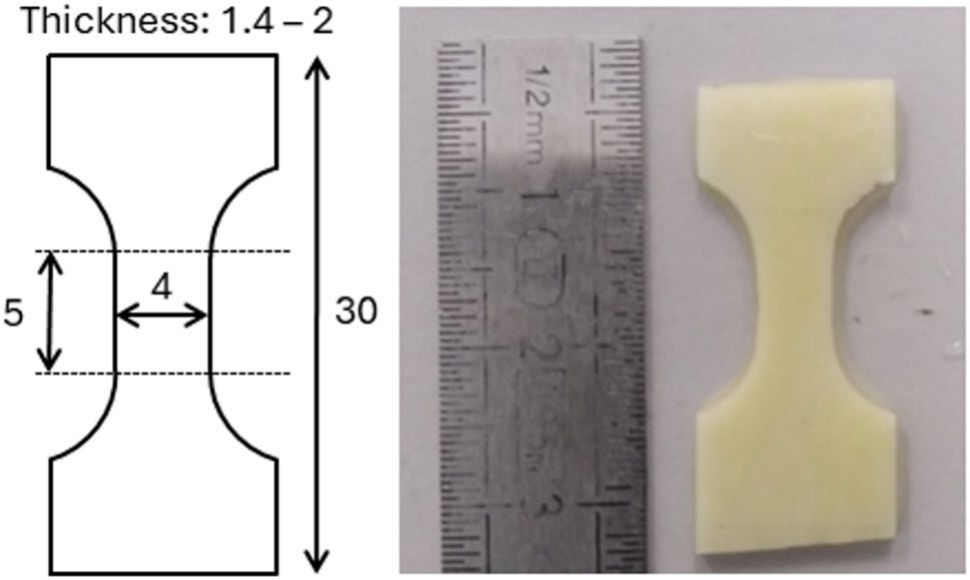
Nominal dimensions and shape of the QS specimens (in mm)

Approximately half the samples were cut with the long axis of the rectangle aligned with the long axis of the bone (longitudinal samples) and the remaining half were cut transversely (transverse samples), see Fig. 1b. The density of each rectangular plate sample was measured by using the measured geometry and its mass which was obtained by weighing the sample on a micro balance. From these, approximately half of each category was machined down further to a dogbone shape according to the dimensions reported in Fig. 2. The IBII specimens were $20 \times 12 \times 2~mm$. For both sets of specimens, exact dimensions were obtained with a calliper.

In total, there were:

- 8 quasi-static longitudinal dogbone specimens;
- 11 quasi-static transverse dogbone specimens;
- 11 longitudinal IBII specimens;
- 8 transverse IBII specimens

The naming convention was as follows: since there were four bones belonging to four different animals, these are referred to as C1 to C4. Quasi-static specimens are identified with QS, while IBII specimens do not have extra labels. Specimens cut from femurs are denoted F while those cut from tibia are denoted T. Finally, each specimen is labelled L or T for longitudinal or Transverse, and they are numbered 1 to 19 for the quasi-static tests and 1 to 19 for the IBII tests. For instance, specimen 12-C1-F-T is an IBII specimen from animal 1, cut from the femur in the transverse direction, and bears number 12 among the IBII specimens.

### Quasi-Static Testing

Samples assigned to quasi-static testing were milled to the specimen geometry already shown in Fig. 2. In order to provide adequate gripping of the samples while limiting stress concentrations, they were shoulder-loaded using a custom laser cut stainless steel grip, the design of which having been performed using finite element analysis. The quasi-static tests were performed on an electromechanical test machine equipped with a 5 *kN* load cell. The samples were loaded in tension under displacement control until failure. Digital Image Correlation (DIC) was used to measure strain on the sample during the test. Details of the full-field measurement set-up are provided later in this section.

### Image-Based Inertial Impact Test Set-Up

The IBII tests were performed using the custom gas gun impact rig described in [35]. The general experimental procedures, imaging and data processing technique for the IBII test are described in detail in [35]. Therefore, the relevant details are only briefly given here.

**Fig. 3:**
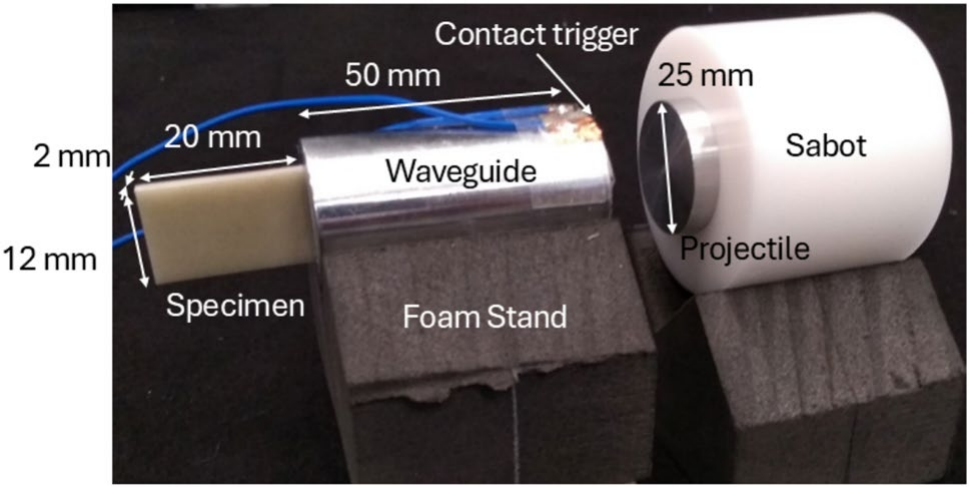
IBII specimen and impactor

A photograph of the IBII test setup is given in Fig. 3. The wave guide and projectile were 25 *mm* diameter cylinders machined from aluminium $Al6061-T6$. The waveguide had a length of 50 *mm* and the projectile had a length of 15 *mm*. The projectile was encased in a 50 *mm* diameter sabot made of delrin. The specimen was attached to the back of the waveguide using cyanoacrylate glue. The waveguide and specimen were mounted on a foam stand so that the front face of the waveguide was aligned to the exit of the gas gun barrel. A copper contact trigger on the front of the waveguide was used to trigger the camera and a light gate assembly in front of the gas gun barrel was used to record the projectile speed and automatically trigger the flash accounting for its rise time.

### Imaging Set-Up and Full-Field Measurement

Previous implementations of the IBII test have used the grid method as a full-field measurement technique because it offers an improved trade-off between spatial and measurement resolution when compared to digital image correlation [36]. This trade-off is especially important when using ultra-high speed cameras with low pixel counts, as is the case for the Shimadzu HPV-X camera used here. A detailed description of the underlying theory of the grid method is not given here but can be found in the review article [37]. The grid pattern was applied by directly printing black squares onto the surface of the samples using the technique described in [35]. For the quasi-static tests, Digital Image Correlation (DIC) was used instead as the low-speed cameras have much more pixels and the tensile tests do no exhibit significant strain gradients. The speckle pattern was applied using an airbrush with black paint, deposited directly onto the specimen as it has a light milky colour so good contrast can be ensured without the need for a white base coat layer. Two Manta G504B cameras were used in a back-to-back configuration with the kinematic data averaged over the two sides, to remove the effect of spurious out-of-plane movement and bending on the measured stiffness components, as detailed in [38].

A summary of the relevant imaging parameters for the quasi-static and dynamics tests is given in Tables 1 and 2. Note that all raw images for the quasi-static and dynamic experiments are given in the digital dataset described at the end of the article. The resolution for the full-field measurement processing chain described below is also given in the appendix.

**Table 1:** Imaging and full-field measurement parameters for the quasi-static tests

Camera	Manta G-504B
Pixel array size	2452 $\times$ 2056
Dynamic range	8 bits
Frame rate	1 fps
Integration (shutter) time	1 ms
Total images	Variable
Lens	Sigma 105 mm
Field of view (FOV)	33.1 $\times$ 27.7 mm$^2$
Measurement technique	2.5D DIC^a^
Software	MatchID 2024.1
Pixel to mm conversion	around 0.08 (slightly different for each specimen)
Average speckle size	0.07 mm
Correlation criterion	ZNSSD
Shape function	affine
Interpolation	Global bicubic spline
Gaussian prefiltering	5x5 pixels
Subset size	31 pixels
Step size	15 pixels
Strain window	3 data points
VSG	61 pixels
Strain shape function	affine
Area of strain averaging	4 $\times$ 4 mm$^2$

^a^2D DIC on each face of the sample

**Table 2:** Imaging and full-field measurement parameters for the dynamic tests

Camera	Shimadzu HPV-X
Pixel array size	400 $\times$ 250
Dynamic range	10 bits
Frame rate	5 Mfps
Integration (shutter) time	110 ns
Total images	128
Lens	Sigma 105 mm
Field of view (FOV)	22.5 $\times$ 14 mm
Measurement technique	Grid method
Grid pitch	0.337 mm
Grid sampling (pxpp)	6 pixels/period^a^
Window type	Bi-triangular
Window width	2pxpp-1
Displacement calculation	iterative
Spatial smoothing	Gaussian, 31 pixels kernel radius
Temporal smoothing	Third order Savitsky-Golay over 21 frames

^a^5 pixels/period for the C3 specimens

### Data Processing

All data processing was performed using in-house programs written in Matlab (version R2019b). The first step in processing IBII data is to obtain displacement fields from the raw experimental images. This was achieved using the grid method with the freely available code^1^ which was made available as part of the review paper in [37]. For the grid method, one grid pitch of displacement data is missing at the edges because the algorithm uses 2N-1 data points where N is the number of pixels sampling a grid period. Previous studies have shown that extrapolation of this missing data improves identifications performed using the VFM [19, 21, 39]. Therefore, the missing displacement data was linearly extrapolated as described in [21].

After extracting the displacement fields, the strain and accelerations fields are obtained by first smoothing the raw displacements and then applying numerical differentiation. All numerical differentiation was performed using a centred finite difference algorithm as implemented by the Matlab function *gradient*. A spatial Gaussian filter was applied to the raw displacements before calculating the strains while a third order temporal Savitsky-Golay filter was applied to the raw displacement before calculating the acceleration. Note that only spatial smoothing was applied to the raw displacements before calculating the strain and only temporal smoothing was applied to the raw displacements before calculating the acceleration.

After obtaining the strain and acceleration fields, they were used along with the VFM identification procedures described in Sect. 2 to obtain the stiffness components and failure stress. When using the stress-gauge equation, stress–strain curves can be constructed at all axial slices of the specimen. Linearly fitting the compressive part of each of these curves gives a single stiffness value meaning that the stiffness can be plotted as a function of position from the free edge (i.e. a function of $x_0$) for each sample. However, as the stress near the free edge is low, the resulting stiffness values are not reliable there. Additionally, edge effects from the spatial smoothing kernel also corrupts the strain data within a half smoothing kernel of the specimen edge. For these reasons the stiffness values obtained over the middle half of the specimen (between $x_0 = 0.25L$ and 0.75*L*) were averaged to obtain a single stiffness value per sample.

A slightly different approach was used for the stress–strain curves generated with the shear stress-gauge. The reason for this is that the shear wave speed is much lower than the longitudinal wave speed so the shear wave did not propagate as far into the specimen. For the specimens with a significant shear response, it was found that it was most stable over the first half of the specimen from the impact edge. Therefore, a single average shear modulus was obtained by averaging over the range $x_0 = 0.5L$ and 0.85*L*. Note that as the shear loading was unintentional, it was not possible to apply this method to all samples.

Unlike the stress–strain curves produced with the stress-gauge, the special optimised virtual fields produce a single stiffness value per time step of the test. At the start of the test, there is not enough strain or acceleration to allow for stiffness identification. Also, during the time at which the stress wave reflects off the free edge and cancels itself, the strains drop to near zero leading to an unstable identification. This produces a temporal stiffness identification which approaches a stable value over the first compressive wave traversal before diverging again. Therefore, a single stiffness value was obtained for each specimen by taking the median response over the temporal range for which the identification was stable. Due to triggering variability, the stable time range for the special optimised virtual fields was selected manually for each specimen. The median was used rather than the mean to avoid the effect of outliers caused by grid defects as discussed in the next paragraph.

The special optimised virtual fields are sensitive to the presence of grid defects (i.e. missing parts of the grid pattern) as described in ref. [21]. For most bone samples, the printed grid pattern did not contain any defects however, on some samples, some of the printed squares debonded. These defects did not have a significant effect on the identification of the primary stiffness component for each sample (i.e. $Q_{11}$ for longitudinal samples and $Q_{22}$ for transverse specimens) but the identification of Poisson’s ratio was corrupted. In order to avoid grid defects corrupting the average stiffness over the stable time steps, the median was used rather than the mean.

A final parameter which needs to be selected for the special optimised virtual fields is the virtual mesh density. Therefore, a virtual mesh refinement study was conducted on synthetic image deformation and experimental data. This showed that low mesh densities where not rich enough to accurately obtain the stiffness components whereas high mesh densities tended to be more sensitive to noise and grid defects. A mesh density of $5 \times 4$ ($x \times y$) elements was found to offer a good compromise. This is the same mesh density that was used for previous IBII tests using the same camera and a similar specimen aspect ratio [19, 21]. The latest version of the IBII processing code which was used to process the experimental data presented in this work is given in the digital dataset described at the end of this article.

## Experimental Results

### Quasi-Static Tests

**Fig. 4:**
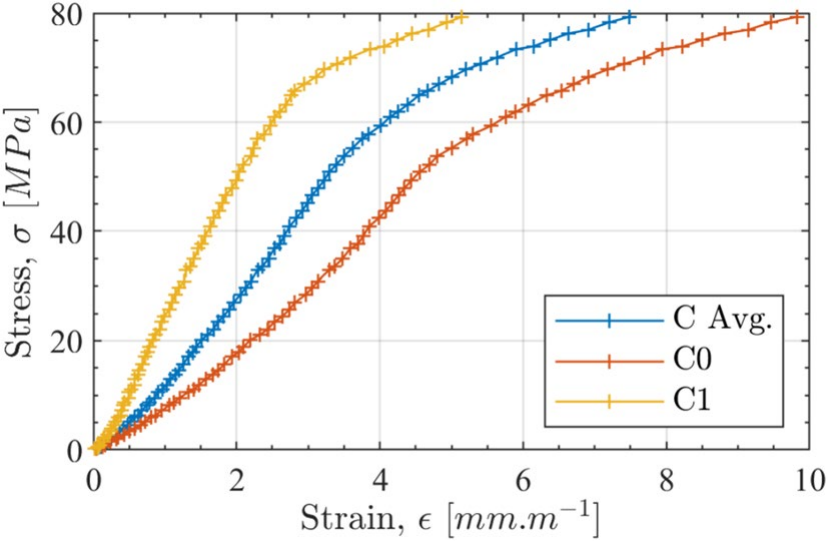
Stress–strain curves for specimen 1-C1-QS-F-L (longitudinal), front, back and averaged

**Fig. 5:**
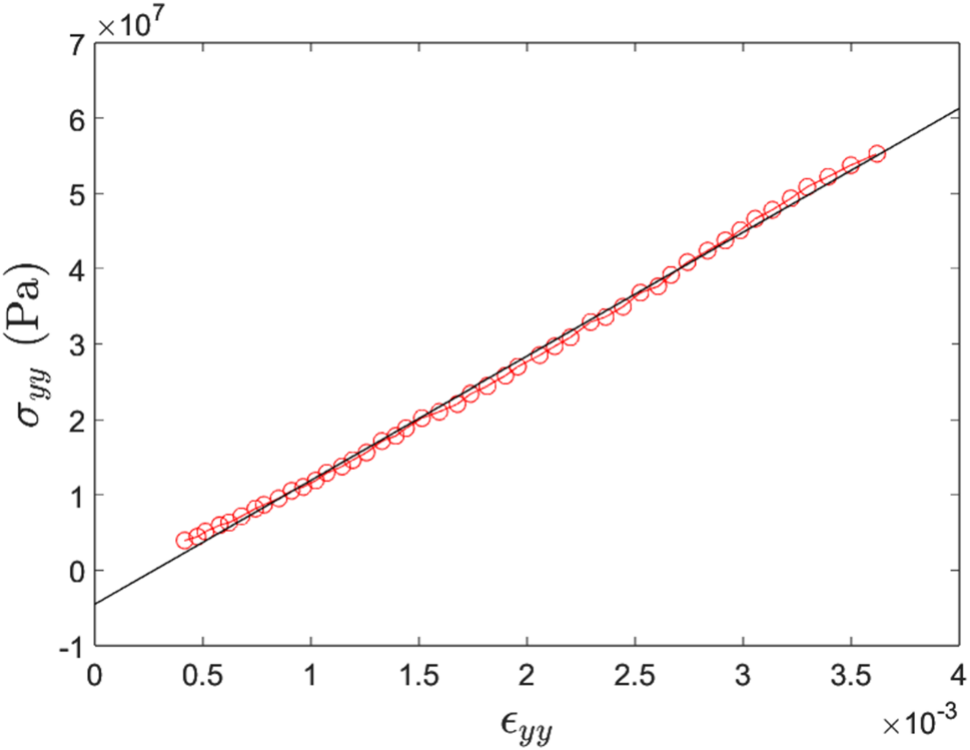
Stress–strain curve in the linear regime for specimen 1-C1-QS-F-L (longitudinal, averaged between front and back faces), with linear fit, Young’s modulus of 13.3 GPa

The longitudinal and transverse strains were averaged over the 5 x 4 mm area shown in Fig. 2. Figure 4 shows an example of stress–strain curves for one of the longitudinal specimens. One can see that the responses over each face are different. This is caused by possible out of plane movement of the specimen as well as potential out of plane bending. The averaged curve compensates for both effects, as already shown in [38]. Figure 5 shows the early, linear part of the average response over which Young’s modulus has been identified. Poisson’s ratio is similarly obtained on the plot of longitudinal vs transverse strains. The results are summarized in Tables 3 and 4. The values of $E_{11}$ are, as expected, higher than those for $E_{22}$ with comparable scatter. The data for $E_{22}$ and $\nu _{21}$ are somewhat more scattered, and in particular, outliers can clearly be seen. This is likely to be related to issues in registering front to back deformation. Using the median rather than the mean, the effect of these outliers can be mitigated. MAD stands for ’Median Absolute Deviation’ and is the counterpart of the standard deviation when the mean is used. COV stands for coefficient of variation and is simply the median normalized by the MAD expressed in percent. For an orthotropic material, we should have the following:

9 $$\begin{aligned} \nu _{21} = \nu _{12}\dfrac{E_{22}}{E_{11}} \end{aligned}$$

Calculating $\nu _{21}$ from $\nu _{12}$, ${E_{11}}$ and ${E_{22}}$, one obtains $\nu _{21}$ = 0.12 which is consistent with the 0.11 from the experiment. This is a good confirmation that the outliers in the transverse tests do not affect the quality of the elastic moduli obtained.

**Table 3:** Summary of the identified quasi-static *E* and $\nu$ for all longitudinal samples

Spec. Num.	$E_{11} [GPa]$	$\nu _{12}$ (–)
1-C1-QS-F-L	13.3	0.14
2-C1-QS-F-L	20.5	0.11
3-C1-QS-T-L	18.9	0.19
4-C1-QS-T-L	21.3	0.22
5-C1-QS-T-L	33.7	0.20
6-C2-QS-F-L	19.2	0.18
7-C2-QS-F-L	21.1	0.18
8-C2-QS-F-L	21.1	0.15
Median	20.8	0.18
MAD	1.05	0.025
COV %	5.0	13.9

**Table 4:** Summary of the identified quasi-static *E* and $\nu$ for all transverse samples

Spec. Num.	$E_{22} [GPa]$	$\nu _{21}$ (-)
9-C1-QS-F-T	14.1	0.05
10-C1-QS-F-T	15.8	− 0.53
11-C1-QS-F-T	14.8	0.11
12-C1-QS-F-T	14.6	0.15
13-C1-QS-T-T	15.4	0.10
14-C1-QS-T-T	8.6	0.11
15-C2-QS-F-T	7.6	0.14
16-C2-QS-F-T	13.9	0.25
17-C2-QS-F-T	15.2	0.11
18-C2-QS-F-T	13.0	0.46
19-C2-QS-F-T	11.6	− 0.65
Median	14.1	0.11
MAD	1.10	0.04
COV %	7.8	36.4

Finally, for all specimens, fracture occurred at the top or bottom fillet of the specimens where the cross-section changes. At this location, significant shear stresses are present and there, fracture does not occur under a state of uniaxial tensile stress. As a consequence, it was decided not to report these values here as they could not be compared to the dynamic ones and would provide a biased dynamic amplification.

### Dynamic Kinematic Fields

**Fig. 6:**
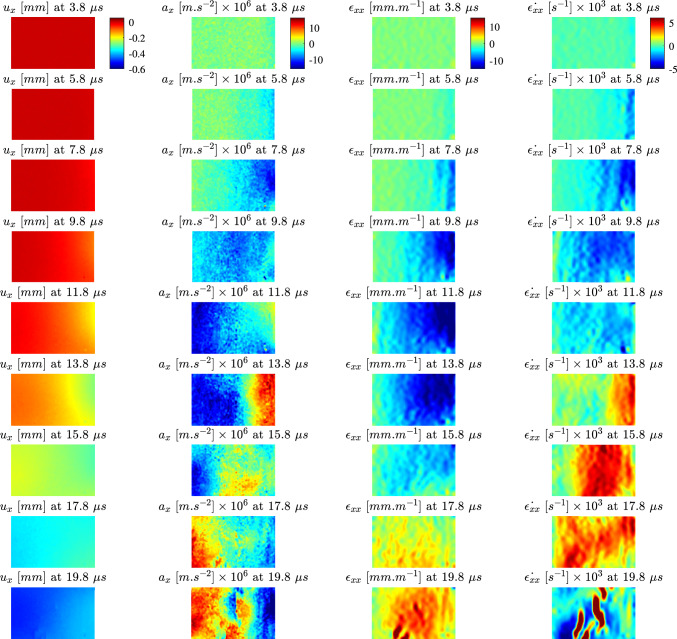
Evolution of the axial kinematic fields for longitudinal specimen 1-C1-F-L. Note the colour bar is constant for all images in the sequence

**Fig. 7:**
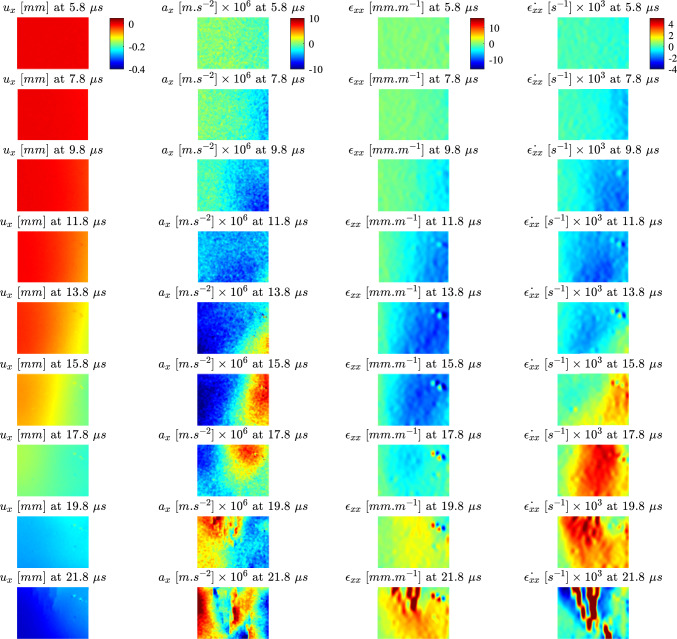
Evolution of the axial kinematic fields for a transverse specimen 15-C2-F-T. Note the colour bar is constant for all images in the sequence

The temporal evolutions of the axial kinematic fields (displacement, acceleration, strain and strain rate) are shown in Fig. 6 for a typical longitudinal specimen and in Fig. 7 for a typical transverse specimen. For both samples, there is an increase in the mean displacement over the test duration with a notable increase after the stress wave reaches the free edge. This is consistent with the rigid body translation expected after the wave reflected.

The wave front is clearly visible in the acceleration and strain fields for both specimens. Peak accelerations for both samples are on the order of $10 \times 10^6~m.s^{-2}$ with the leading edge of the impact pulse producing a band of negative acceleration which propagates across the sample. Initially, the strains in both samples are negative as the first compressive wave travels across the sample to the free edge. After wave reflection, there is a build up of tensile strain which causes the sample to fail in tension. This results in a multiple cracks in the region of high tensile strain which can be observed as non-physical concentrations in the strain fields.

In Figs. 6 and 7, it is shown that the strain rate for the IBII test is heterogeneous. As the loading occurs over such a short duration in the IBII test (on the order of microseconds), it is unlikely that any long term viscous mechanisms have time to contribute to the response. Thus, while the strain varies from zero to a value on the order of several thousand $s^{-1}$, the test can be best represented using an indicative strain rate measure. Therefore, we define the effective strain rate as follows:

10 $$\begin{aligned} \hat{\dot{\epsilon _{ij}}} =\frac{\sum ^{N} \sum ^{t} \epsilon _{ij} \dot{\epsilon _{ij}}}{\sum ^{N} \sum ^{t}\epsilon _{ij}}~~,~~i,j=x,y \end{aligned}$$

where $\hat{\dot{\epsilon _{ij}}}$ is the effective strain rate and the summations are performed over *N* measurement points in space and *t* time steps. This metric is used in the following section to indicate the strain rate for stiffness identification and failure stress analysis.

### Dynamic Stiffness Identification

This section begins with the results for stiffness identifications performed with the special optimised virtual fields followed by the identifications with the stress-gauge. The final part of this section gives a summary of the identified stiffness and a comparison to recent data in the literature.

**Fig. 8:**
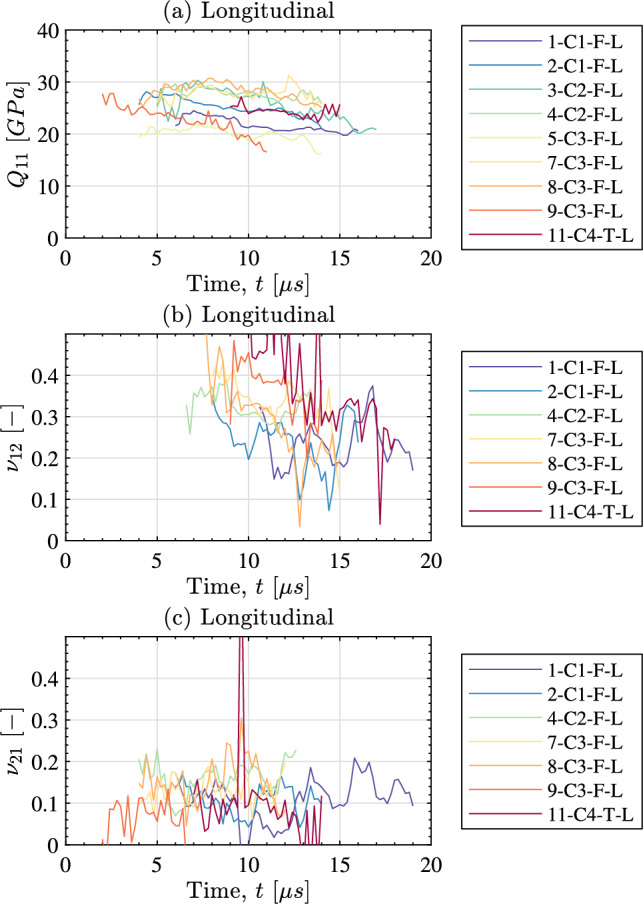
Identified stiffness as a function of time for the longitudinal samples when using the special optimised virtual fields. Samples with an unstable response due to grid defects have been omitted (these are shown as a ‘–’ in Table 5)

**Fig. 9:**
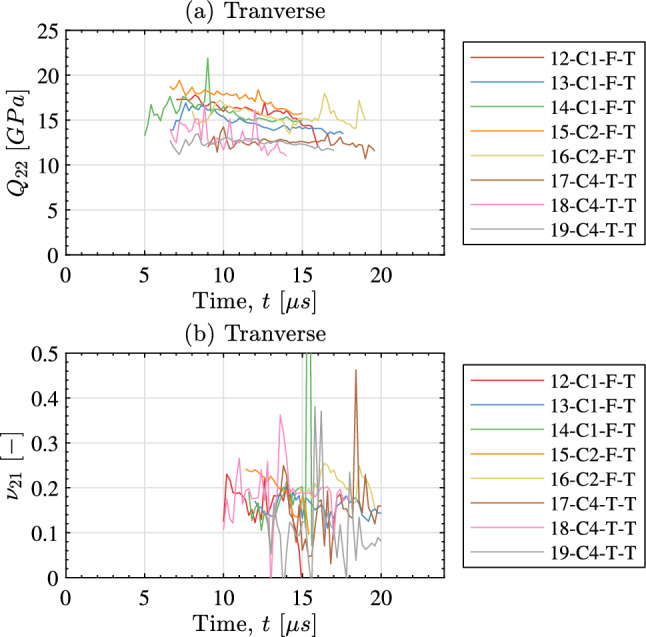
Identified stiffness as a function of time for the transverse samples when using the special optimised virtual fields. Samples with an unstable response due to grid defects have been omitted (these are shown as a ‘–’ in Table 6)

The identified stiffness parameters using the special optimised virtual fields are shown in Fig. 8 for the longitudinal samples and in Fig. 9 for the transverse samples. As previously mentioned, the special optimised virtual fields give the identified stiffness as a function of time with the data shown in Figs. 8 and 9 only showing frames where the response is stable. For both sample orientations it is clear that the stiffness component aligned with the impact direction is the easiest to identify. It is much more difficult to temporally resolve the Poisson’s ratio for both cases. Typically, the Poisson’s ratio is extremely difficult to obtain as the lateral strains are small and have a low signal to noise ratio. For the aligned impact case considered here, this parameter is weakly activated. An additional difficulty occurs due to presence of grid defects (i.e. missing parts of the grid pattern). This caused the identification to be unstable for some specimens so these results have been removed from Fig. 8. The same problem has been observed previously for through-thickness carbon fibre composite samples as described in [21]. Unfortunately, the shear response was not strong enough to allow for accurate identification of the shear modulus with the special optimised virtual fields for either the longitudinal or transverse samples. However, for some specimens it was possible to obtain the shear modulus using stress–strain curves constructed with the shear stress-gauge equation, as described later in this section. For the special optimised virtual fields, a single stiffness parameter was obtained by taking the median over the stable temporal range shown in Fig. 8. The median identified stiffness for each sample is summarised in Table 5 for the longitudinal samples and in Table 6 for the transverse samples.

**Fig. 10:**
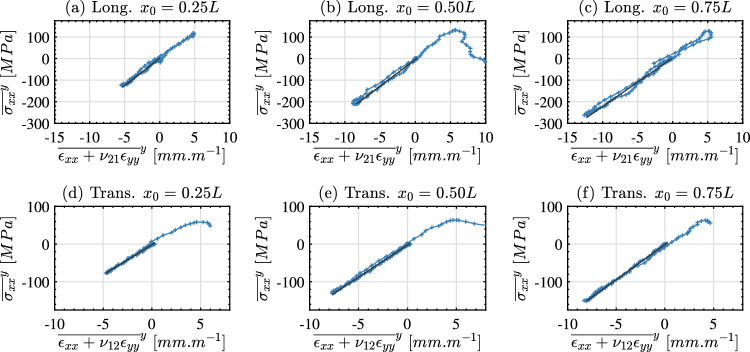
Stress–strain curves at three locations along the specimen length (x_0_ = 0.25, 0.5, 0.75 L) for the longitudinal sample 1-C1-F-L (**a**)–(**c**) and for the transverse sample 15-C2-F-T (**d**,** e**). The fitted response used to obtain the relevant stiffness component is shown using a solid black line

Next, we present the stiffness identification performed using stress-gauge equation beginning with the stress–strain curves for a representative longitudinal and transverse specimen. In order to construct stress–strain curves for stiffness identification, it is necessary to know the relevant Poisson’s ratio to calculate the average strain. As it was not possible to obtain the Poisson’s ratios for each individual sample due to grid defects, the average identified Poisson’s was used ($\nu _{12} = 0.31$ and $\nu _{21} = 0.16$). A series of stress strain curves at 25, 50 and 75% of the sample length are shown in Fig. 10 for a longitudinal and transverse sample. Both samples exhibit a linear elastic load and unload in compression with the peak average stress experienced by the longitudinal sample being higher than the transverse. For both samples, the stress–strain curve at $x_0 = 0.50L$ shows unloading in tension with minimal non-linearity before the peak stress is reached and the sample fails. Extraction of the tensile failure stress is considered in the following section but for now we focus on extracting the stiffness from the stress–strain curves by linearly fitting the compressive part of the curve.

**Fig. 11:**
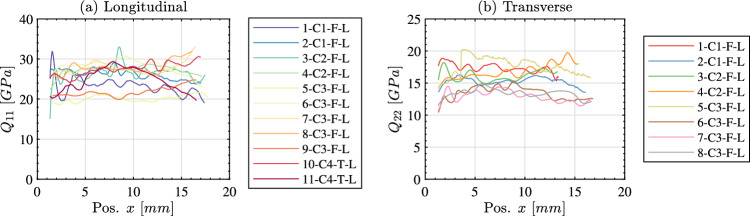
Stiffness identified by linearly fitting the stress–strain curves as a function of position from the free edge. All longitudinal samples are shown in (**a**) and all transverse samples are shown in (**b**)

Linearly fitting all stress–strain curves along the length of each sample produces a plot of the identified stiffness as a function of position (i.e. $Q_{11}$ for the longitudinal samples and $Q_{22}$ for the transverse samples). This is shown in Fig. 11a for the longitudinal samples and Fig. 11b for the transverse samples. In this figure, data that is corrupted by smoothing edge effects has been removed. Specifically, data within a grid pitch plus half a spatial smoothing kernel ($6+31~pixels$ or 2.08 *mm*) of data points were removed from each edge. For the longitudinal samples the $Q_{11}$ stiffness is typically within the bounds of 20 to 30 *GPa* whereas the $Q_{22}$ of the transverse samples is lower being typically between 10 to 20 *GPa*. A single stiffness value was extracted for each specimen by averaging over the middle 50% of the specimen length (between $x_0 = 0.25L$ and $x_0 = 0.75L$). The resulting stiffness for all longitudinal samples is given in Table 5 and for all transverse samples in Table 6.

**Fig. 12:**
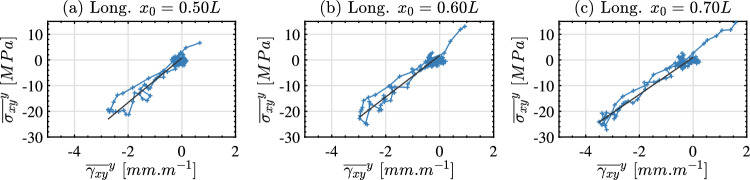
Shear stress–strain curves for sample 7-C3-F-L. The fitted response used to obtain the shear stiffness is shown as a solid black line

**Fig. 13:**
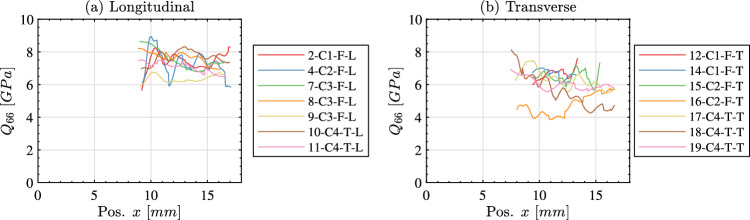
Shear stiffness identified by linearly fitting the stress–strain curves as a function of position from the free edge. Longitudinal samples are shown in (**a**) and transverse samples are shown in (**b**). Only samples that had a significant shear response are shown; omitted samples are denoted with a ‘–’ in Tables 5 and 6

For some samples, the shear response was significant enough to obtain shear stress–strain curves using the shear stress-gauge equation (Eq. 7). The shear stress–strain curves for a longitudinal sample are shown in Fig. 12. The response in shear is much noisier than that observed in the loading direction with much lower peak strains. Additionally, the stress–strain curves in Fig. 12 show that the peak shear strain decays significantly along the specimen length with the stress–strain curve at x_0_ = 0.5 L being much noisier than the curves near the impact edge. For this reason, it was only possible to obtain the shear stiffness as a function of position between x_0_ = 0.5 L and the impact edge (excluding smoothing edge effects as before). The shear stiffness as a function of position from the free edge is shown in Fig. 13a for the longitudinal samples and in Fig. 13b for the transverse samples. A single shear stiffness value was obtained for each sample by averaging over the range x_0_ = 0.5 L and x_0_ = 0.85 L. The shear stiffness results are summarised in Table 5 for the longitudinal samples and Table 6 for the transverse samples. Samples that did not exhibit a significant shear response are excluded from the tables.

**Table 5:** Summary of the identified stiffness components for all IBII longitudinal samples

Longitudinal			Opt. VFs			Stress-Gauge	
Spec. Num.	Density $\rho$ $[kg.m^{-3}]$	$\hat{\dot{\epsilon _{xx}}}$ $[s^{-1}]$	$Q_{11}$ [*GPa*]	$\nu _{12}$ $[-]$	$\nu _{21}$ $[-]$	$Q_{11}$ [*GPa*]	$Q_{66}$ [*GPa*]
1-C1-F-L	1980	− 2086	21.3	0.23	0.11	23.54	–
2-C1-F-L	2028	− 1963	25.0	0.25	0.11	26.52	7.16
3-C2-F-L	2118	− 1147	25.2	–	–	27.04	–
4-C2-F-L	2118	− 1999	27.7	0.31	0.17	27.76	7.62
5-C3-F-L	1934	− 1149	20.0	–	–	20.13	–
6-C3-F-L	2038	− 1513	–	–	–	29.07	–
7-C3-F-L	2032	− 1027	27.8	0.34	0.13	27.58	7.77
8-C3-F-L	2031	− 1284	28.4	0.31	0.15	27.36	7.79
9-C3-F-L	1904	− 1018	23.0	0.38	0.07	21.47	6.28
10-C4-T-L	2118	− 846	–	–	–	27.73	7.54
11-C4-T-L	1991	− 1105	24.4	0.34	0.09	26.87	7.15
Median	2031	− 1149	25.0	0.31	0.11	27.0	7.54
MAD	51	134	2.69	0.03	0.02	0.68	0.25
COV %	2.5	11.7	10.8	9.0	19.9	2.5	3.3

**Table 6:** Summary of the identified stiffness components for all IBII transverse samples

Transverse			Opt. VFs		Stress-Gauge	
Spec. Num.	Density $\rho$ $[kg.m^{-3}]$	$\hat{\dot{\epsilon _{xx}}}$ $[s^{-1}]$	$Q_{22}$ [*GPa*]	$\nu _{21}$ $[-]$	$Q_{22}$ [*GPa*]	$Q_{66}$ [*GPa*]
12-C1-F-T	1911	− 1403	16.3	0.16	17.3	6.9
13-C1-F-T	1961	− 1557	14.4	0.16	15.4	–
14-C1-F-T	1964	− 1255	15.6	0.16	15.8	6.8
15-C2-F-T	2043	− 1268	17.8	0.20	16.6	6.6
16-C2-F-T	2040	− 2569	15.3	0.21	18.3	4.6
17-C4-T-T	1853	− 933	12.5	0.15	14.0	6.3
18-C4-T-T	1879	− 1354	12.7	0.19	13.2	5.8
19-C4-T-T	1866	− 1257	12.4	0.10	13.7	6.1
Median	1936	− 1311	14.8	0.16	15.6	6.33
MAD	64	74	1.78	0.02	1.63	0.49
COV %	3.3	5.6	12.0	11.0	10.4	7.8

### Discussion on Stiffness Identification

Having presented the stiffness identification results for each method, it is useful to analyse the overall data set as presented in Tables 5 and 6. For the special optimised virtual fields, the average longitudinal stiffness over all samples was 25.0 *GPa* at an effective strain rate on the order of $1000~s^{-1}$. As expected, this is higher than the transverse stiffness which was 14.8 *GPa* at approximately the same effective strain rate. The average major Poisson’s ratio is 0.31 whereas the average minor Poisson’s ratio was 0.11 for the longitudinal samples. Only the minor Poisson’s ratio was able to be obtained for the transverse samples as the image deformation simulations predicted an extremely high total error (on the order of $50\%$). The average minor Poisson’s ratio for the transverse samples was 0.16. The data in Tables 5 and 6 shows that obtaining the dominant stiffness component (i.e. $Q_{11}$ for the longitudinal samples and $Q_{22}$ for the transverse samples) with either the optimised virtual fields or the stress-gauge equation produces consistent results. For both cases, the median stiffness identified with the special optimised virtual fields is within one median absolute deviation of the median for all samples when the identification is performed with the stress-gauge.

Given that the material properties of bone vary based on biological factors such as sample location, and donor variability, higher scatter was expected compared to traditional engineering materials. And indeed, the variability in the identified stiffnesses is high compared to previous studies using the IBII test on carbon fibre composites, see for example [21, 22, 34, 40]. Compared to the quasi-static tests in Sect. 4.1, the variability is in fact slightly lower for the IBII tests, consistent with the fact that the boundary conditions are simpler, no gripping, and alignment is performed using the thorough procedure proposed in [40]. Unfortunately, the IBII test is destructive so it is not possible to repeat the test multiple times on a single sample to analyse test-to-test variability. In the future, it should be possible to combine different image-based test methods to span several orders of magnitude in strain rate on the same test sample isolating the rate dependence from specimen-to-specimen variability. This is discussed further in Sect. 5.

To compare dynamic and quasi-static results, the median values from Tables 3, 4, 5 and 6 are summarized in Table 7. For $Q_{11}$ and $Q_{22}$, the average of the values obtained with optimized virtual fields and stress-gauge equation are reported, and for $\nu _{21}$, the average between longitudinal (0.11) and transverse (0.16) is reported. As expected, the stiffness components $Q_{11}$ and $Q_{22}$ exhibit a positive strain rate dependence, even though the effect is more prominent on $Q_{11}$ than on $Q_{22}$. This dependence is broadly in line with the data from [5]. Human bone exhibits a number of compositional and micro-structural differences that would lead to differences in the magnitude of overall rate sensitivity compared to bovine bone. However, the overall trends and magnitudes should be similar as fundamental components of bone are similar (i.e. collagen, calcium hydroxyapatite and water).

For Poisson’s ratios, the scatter is such that it is very difficult to draw any conclusion. There is a large difference on the values of $\nu _{21}$ obtained between the longitudinal (0.11) and the transverse (0.16) samples. Moreover, when calculating $\nu _{21}$ from $E_{11}$, $E_{22}$ and $\nu _{12}$ using Eq. 9, the quasi-static data are consistent but the dynamic data, not so much. It looks like $\nu _{12}$ is overestimated in the IBII test, the $\nu _{21}$ value obtained from Eq. 9 is 0.18 instead of 0.13 (which is the average of 0.11 and 0.16). This is something that will require future investigation to understand this discrepancy, as well as the difference between $\nu _{21}$ from the longitudinal and transverse directions.

**Table 7:** Summary of identified elastic constants over quasi-static (QS) and dynamic (IBII) tests. Dyn. Ampl. is the dynamic amplification factor providing the relative difference between high rate and quasi-static values. *Recalculated from the three other components using Eq. 9

Stiffness component	IBII	QS	Dyn. Ampl. (%)
$Q_{11} [GPa]$	26	21.2	22
$Q_{22} [GPa]$	15.2	14.4	5.5
$\nu _{12} [-]$	0.31	0.18	72
$\nu _{21} [-]$	0.13	0.11	23
$\nu _{21}* [-]$	0.18	0.12	–

Unfortunately, it was not possible to obtain the shear modulus for the quasi-static test data in this study. However, a recent study used DIC to analyse the shear modulus of bovine bone using an Arcan type test rig [41], so this could be used in a future testing campaign together with shear-dominated IBII tests (see Sect. 5).

### Dynamic Failure Stress Identification

This section begins by presenting the process for obtaining tensile failure stress for the sample 1-C1-F-L to outline the procedure.

**Fig. 14:**
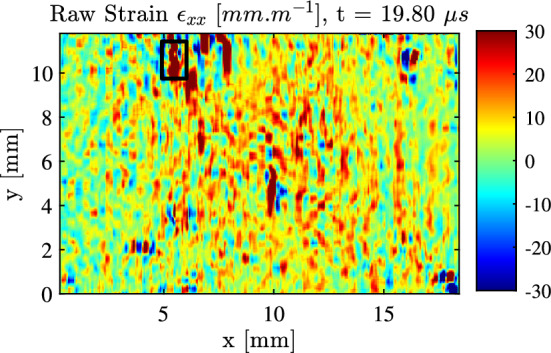
Raw strain map (no smoothing applied) for sample 1-C1-F-L showing the identified fracture location and virtual gauge area with a black rectangle

The first step in the tensile failure stress identification procedure is to identify the first fracture location using strain maps derived from unsmoothed displacements. This is shown in Fig. 14 for sample 1-C1-F-L. Here the fracture location is observed as a region of unphysical tensile strain concentration (in reality, crack opening) which propagates as the sample fails. Then, over a small area encompassing this fracture location and represented as a black rectangle in Fig. 14, two metrics of stress have been calculated, both denoted with the superscript VG standing for virtual gauge. First, since the stiffness components have been identified for each specimen, stress scan be reconstructed from strain and an average stress from strain calculated over that small area. Secondly, using the linear stress gauge (LSG) relationship expressed in Eq. 8 from Sect. 2.3, a linear approximation of the longitudinal stress as a function of the vertical coordinate can be calculated for each cross-section; and this stress metric deriving solely from acceleration can be averaged over the same small area and compared to the stress-from-strain one. In absence of fracture, these two metrics should match as long as the stress distribution along each cross-section is linear. Then when fracture occurs, the stress obtained from strain shoots up as the strains are not physical anymore, and this allows a reading of the local failure stress.

**Fig. 15:**
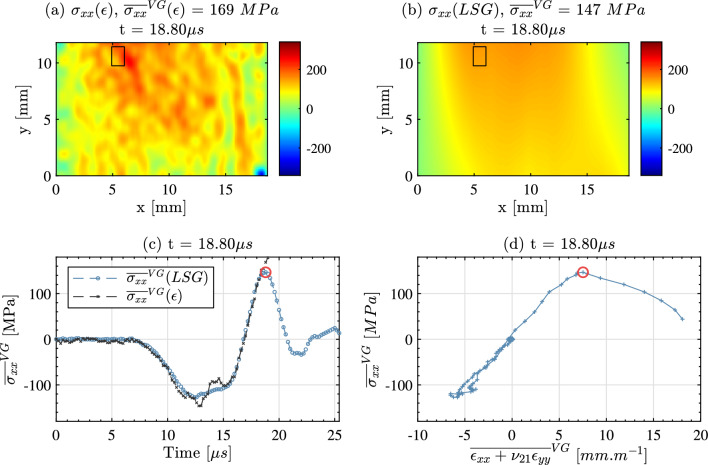
Failure stress identification diagnostics for sample 1-C1-F-L. Axial stress field calculated from the strain (**a**) and axial stress field from the linear stress gauge (**b**) with the virtual stress gauge shown as a black rectangle. Comparison of stress measures as a function of time (**c**) and local stress–strain curve over the virtual stress gauge area (**d**) with the time at which the failure stress was extracted shown as a red circle

This process is illustrated in Fig. 15. The top row shows the stress maps for the two approaches, stress from strain ((a), left) and stress from acceleration through the LSG equation ((b), right). The two maps are provided at $18.8~\mu s$, the time just before fracture. One can see a good similarity between the two. On the bottom row left, figure (c), the temporal evolution of the average of the stresses from the two metrics is provided. One can see a good correspondence between the two in the compressive part, some deviation at the rebound with some oscillations in the strain-derived stress and then, again, good correspondence in the unloading then tensile loading part until a sharp separation of the two occurs at $18.8~\mu s$. At this point, the failure stress was identified from the LSG map as 147 MPa. The right hand-side plot on the bottom row, (d), shows the local (VG) stress–strain curve from the acceleration-derived stress, showing a slope change in the response because of the fracture.

The failure stress results are reported in Tables 8 and 9, together with the effective strain rates (all on the order of several thousands $s^{-1}$). The median for the longitudinal failure stress is 146 MPa and only 58.35 MPa in the transverse direction, showing a strong strength anisotropy

**Table 8:** Effective strain rate and failure stress for all longitudinal samples that fractured during the test

Longitudinal		
Spec. Num.	$(\hat{\dot{\epsilon _{xx}}})^{VG}$ $[s^{-1}]$	$\overline{\sigma _{xx}(LSG)}^{VG}$ [*MPa*]
1-C1-F-L	5410	146.6
2-C1-F-L	6889	145.8
3-C2-F-L	4020	106.5
4-C2-F-L	5206	116.8
6-C3-F-L	2365	156.7
Median	5206	145.8
MAD	1186	10.9
COV %	23	7.5

**Table 9:** Effective strain rate and failure stress for all transverse samples that fractured during the test

Transverse		
Spec. Num.	$(\hat{\dot{\epsilon _{xx}}})^{VG}$ $[s^{-1}]$	$\overline{\sigma _{xx}(LSG)}^{VG}$ [*MPa*]
12-C1-F-T	4985	58.3
13-C1-F-T	4985	67.3
14-C1-F-T	3486	78.0
15-C2-F-T	5455	62.8
16-C2-F-T	6173	54.8
18-C4-T-T	4022	45.4
19-C4-T-T	3086	53.6
Median	4985	58.3
MAD	963	4.7
COV %	19	8.1

### Comparison with Existing Data

It is difficult to compare the current set of data with existing results in the literature as most high strain rates studies of bone are in compression ([5, 10, 42–44], for instance). While compressive loading may yield moduli values relevant to tensile loading [45], failure occurs by instability and is more representative of a structural behaviour than an intrinsic material property. The reason why compression is often used for strain rates above 100 $s^{-1}$ is that for such strain rates, the Split Hopkinson Pressure Bar (SHPB) is mostly used and is much easier to perform in compression [5, 43]. In that case, small block-like samples can be used and the specimens are simply positioned between the input and output bars, without the need for any specific clamping fixture. There are a few studies reporting tensile tests at moderate [46] to high [6, 47–49] strain rates. Table 10 summarizes data from some of the above references to compare with the data obtained in the present paper. It can be seen that our data aligns broadly with that from literature. It should be noted however that our data is obtained at much higher strain rates than available in the literature. In fact, there is little to no tensile moduli and fracture stresses at such strain rates as reported in the present paper.

**Table 10:** Comparison of high rate longitudinal properties of bovine bone with literature

Reference	Test	$E_{11}$ (GPa)	Fracture stress (MPa)	Strain rate ($s^{-1}$)
[42]	Compression	42	–	1500
[47]	Tension	13.0	250	250
[46]	Tension	23.4	140	0.15
[6]	Tension	40.4	271	237
[43]	Compression	14.1	–	0.15
[48]	Tension	21.1	–	1
[49]	Tension	14.9	121	300
[50, 51]	Tension	10–21.7	33–50	15*
[10]	Compression	15.9	–	120
Present work	IBII (tensile fracture)	25.1	146	5200

*Evaluated from specimen dimensions and cross-head speed

## Limitations and Future Work

The results of this study provide new data for the rate dependence of the stiffness and tensile failure stress of cortical bone, at strain rates higher than reported in previous studies. Such strain rates may be representative to local strain rates at strain concentrators (wedge impact) during fracture, or more extreme cases like ballistic perforation or blast. The IBII test method provides high quality data in the elastic domain compared to standard Split Hopkinson Bar tests but it is quite new so it is important to understand its limitations and where future effort should concentrate.

**3D effects** one of the key assumptions when applying the VFM to IBII test data is the requirement that the test is two dimensional such that the surface full-field measurements are representative of through-thickness behaviour and the three dimensional integrals in the principle of virtual work collapse to two dimensions. A recent study investigated this problem in detail using back-to-back imaging [40]. In this study it was found that the stiffness identification procedure was not strongly influenced but that the local stress derivation through the linear stress gauge was more affected, leading to up to 30% bias on the failure stress. The new alignment procedure proposed in [40] allows to mitigate these biases.

**Stiffness identifiability** this study was focused on obtaining stiffness and tensile failure stress data for bone under high strain rate loading. In order to obtain the tensile failure stress, it was necessary to test samples in the two primary orientations (i.e. longitudinal and transverse). This aligned impact configuration is far from optimal in terms of providing enough heterogeneity to extract all stiffness components of a cortical bone sample in a single test. In the spirit of Material Testing 2.0 [52], it is possible to extend the IBII test to more heterogeneous IBII configurations.

In [34] off-axis carbon fibre samples were tested and the transverse and shear modulus were obtained. However, for carbon fibre composites, the fibre stiffness is not rate sensitive and is much larger than the transverse stiffness (by an approximate factor of 10). This meant that the rate sensitivity of the transverse and shear response could be obtained without needing the remaining two orthotropic stiffness components. The problem becomes much more interesting for materials such as glass fibre composites and bone which have have longitudinal and transverse stiffness which are more similar (approximately a factor of 4 difference for glass fibre and a factor of 2 for bone). Therefore, it would be interesting to try the off-axis IBII test for bone. It is worth noting however that failure is likely to occur under a combined stress state, requiring a failure criterion to interpret the results.

In [25], samples with notches and holes were used to increase the heterogeneity of IBII test for visco-plasticity identification. In that spirit, introducing strain concentrators in the specimen design, combined with off-axis material orientation, could provide much richer stress and strain states to allow for the full identification of the in-plane stiffness tensor. However, the limited spatial resolution of the Shimadzu HPV-X camera has the potential to lead to systematic errors in the strain field reconstruction, which would need to be assesed using synthetic image deformation as in [39].

For the near future, a good starting point would be to use a combination of impacting over some portion of the sample height and combine this with an off-axis sample removing the need to introduce holes or notches. The concept of a half-height impact was used to validate the generalized stress–strain curve procedure discussed in [33]. The half-height impact test has not been applied experimentally yet but is a promising avenue for enriching the kinematics of the IBII test and provide the full set of in-plane orthotropic stiffness components in a single test.

**Identification of heterogeneous properties** in this study, the individual samples were treated as homogeneous and the material properties were identified for each individual sample. However, bone is a heterogeneous material with properties that vary in space. The use of image-based measurements (such as DIC or the grid method) provides new opportunities for exploring spatial stiffness distributions. The VFM has been used in the past to identify elastic stiffness distributions [53–56] but one of the main challenges under high rate loading is limited number of pixels available on the current generation of ultra-high speed cameras (only $400 \times 250~pixels$ for the Shimadzu HPV-X used here). Combining image-based measurements, inverse identification procedures and micro-CT information could provide wholly new information on the accuracy and validity of density based material property assignment strategies for micro-CT based finite element models.

**Interfaces** the IBII test could be adapted to test the adherence between bone and bone cement. In the past, another image-based high strain rate test, the Image-Based Ultrasonic Shaking (IBUS) test, has been used to test bone [57] and bone cement [58] but is not capable of fracturing the specimens, so the IBII test could investigate fracture of bone, bone cement and interfaces between the two in the same spirit as the initial work performed with the IBII test on adhesive bonding [59].

**IBIR test** one of the limitations of the IBII test is that only high rate stiffness components can be identified on a single specimen, even though all stiffness components could potentially be identified on the same specimen in the future. Therefore, quasi-static stuffness components have to be measured on a different specimen, mixing spatial variability of the properties and strain rate effects. The recently developed Image-Based Inertial Release (IBIR) test [60] has the potential to obtain quasi-static and high rate stiffness components on the same specimen, allowing complete disentanglement of the strain rate sensitivity and spatial scatter. A specimen like that used in [61] could be employed in the IBIR test. This is an interesting perspective which would increase understanding of the mechanical properties of human bone and make the most of available tissue samples.

## Conclusion

This study provides new data for the constitutive behaviour of bovine bone at high strain rates using the IBII test method. The focus of this work was the accurate identification of the orthotropic stiffness parameters under high strain rate loading as well as the tensile failure stress. The method relies of inertially impacting thin rectangular specimens oriented longitudinally or transversally to the bone shaft. The underpinning idea is to use the acceleration field measured through ultra-high speed imaging of a deforming grid as a volume distributed load cell. The main conclusions of this work are summarised as follows:

1. The average longitudinal stiffness was found to be 26 *GPa* at an effective strain rate of $1150~s^{-1}$ and the transverse stiffness was found to be 15.2 *GPa* at an effective strain rate of $1300~s^{-1}$. Using paired quasi-static samples, this represents a $22\%$ rate sensitivity for the longitudinal samples and a $5.5\%$ rate sensitivity for the transverse samples.
2. Slight misalignments of the projectile made it possible to obtain the shear modulus for some samples with an average shear modulus over all samples of 6.9 *GPa* with an effective strain rate of $1200~s^{-1}$.
3. The average tensile failure stress of the longitudinal samples was 146 *MPa* at a strain rate of $5200~s^{-1}$. The average tensile strength of the transverse samples was 53.6 *MPa* at a strain rate of $5000~s^{-1}$.
4. The stiffness and failure stress values broadly align with data in the literature, though the IBII test allows to reach much higher strain rates as it is not limited by the quasi-static assumption required to analyse SHB tests.
5. Using the compression wave reflection to create tensile fracture (as in a spalling test) leads to much simpler boundary conditions, resulting is rather low scatter of the data for such biological tissues.
6. Unintended in-plane impact misalignment generated shear waves allowing for the shear modulus to be identified. In the future, systematic misalignment should be introduced to have consistent data for all tested specimens.

The use of image-based test methods has significant potential in the area of bone bio-mechanics. Biological materials are inherently complex in terms of composition and structure leading to anisotropic and heterogenous properties. The use of image-based test methods provides a wealth of data which can be used to obtain information for material models of bone that was not previously possible.

## Data Availability

All data supporting this study are openly available from the following repository at: https://zenodo.org/uploads/14628655. The digital dataset contains the following: 1. Raw image data for the quasi-static and dynamic tests including static images used for calculating the measurement resolution. 2. Matlab codes used for processing the IBII test data. 3. All output from the Matlab processing code including image sequences of all kinematics and strength diagnostics for the IBII tests.

## Appendix: Measurement Resolution

### Quasi-Static Tests

See Tables 11, 12.

**Table 11:** Measurement resolution for the quasi-static tests (longitudinal samples)

	u$_x$ [$\mu$m]	u$_y$ [$\mu$m]	$\varepsilon _{xx}$ [$\mu \varepsilon$]	$\varepsilon _{yy}$ [$\mu \varepsilon$]	$\varepsilon _{xy}$ [$\mu \varepsilon$]
1-C1-F-L					
Camera 0	0.073	0.089	2.71	6.98	4.05
Camera 1	0.10	0.10	25.6	14.3	14.5
2-C1-F-L					
Camera 0	0.12	0.18	6.25	4.51	3.22
Camera 1	0.13	0.10	15.7	3.9	6.70
3-C2-F-L					
Camera 0	0.13	0.12	24.9	12.4	13.2
Camera 1	0.10	0.10	25.6	14.3	14.5
4-C2-F-L					
Camera 0	0.08	0.09	17.2	16.3	12.2
Camera 1	0.09	0.09	14.0	6.20	7.73
5-C3-F-L					
Camera 0	0.40	0.42	3.40	24.7	13.8
Camera 1	0.10	0.13	7.10	2.87	3.83
6-C3-F-L					
Camera 0	0.15	0.14	62.2	7.15	22.5
Camera 1	0.13	0.20	19.2	17.3	9.51
7-C3-F-L					
Camera 0	0.12	0.11	28.2	4.14	11.3
Camera 1	0.09	0.10	11.9	3.54	4.56
8-C3-F-L					
Camera 0	0.19	0.16	6.21	5.29	20.9
Camera 1	0.13	0.17	9.13	5.87	5.55
Mean	0.13	0.14	17.4	9.36	10.5

**Table 12:** Measurement resolution for the quasi-static tests (transverse samples)

	u$_x$ [$\mu$m]	u$_y$ [$\mu$m]	$\varepsilon _{xx}$ [$\mu \varepsilon$]	$\varepsilon _{yy}$ [$\mu \varepsilon$]	$\varepsilon _{xy}$ [$\mu \varepsilon$]
9-C1-QS-F-T					
Camera 0	0.06	0.06	2.46	3.50	1.93
Camera 1	0.12	0.12	19.0	9.67	10.0
10-C1-QS-F-T					
Camera 0	0.09	0.11	4.34	4.70	3.46
Camera 1	0.07	0.08	3.95	4.50	4.49
11-C1-QS-F-T					
Camera 0	0.10	0.13	3.22	5.53	3.18
Camera 1	0.13	0.15	7.71	8.42	7.67
12-C1-QS-F-T					
Camera 0	0.14	0.11	4.75	5.62	5.62
Camera 1	0.08	0.07	4.25	2.87	2.47
13-C1-QS-T-T					
Camera 0	0.08	0.08	2.78	4.93	4.73
Camera 1	0.14	0.14	6.58	8.42	5.96
14-C1-QS-T-T					
Camera 0	0.44	0.43	16.1	36.7	25.0
Camera 1	0.18	0.18	14.4	11.8	8.23
15-C2-QS-F-T					
Camera 0	0.14	0.10	36.6	4.27	12.7
Camera 1	0.10	0.12	5.38	4.38	4.02
16-C2-QS-F-T					
Camera 0	0.14	0.12	53.9	6.09	20.9
Camera 1	0.07	0.07	18.6	4.15	7.19
17-C2-QS-F-T					
Camera 0	0.09	0.08	7.23	3.85	4.19
Camera 1	0.08	0.09	3.35	4.22	2.83
18-C2-QS-F-T					
Camera 0	0.10	0.10	9.43	8.85	7.21
Camera 1	0.10	0.11	4.51	5.79	3.58
19-C2-QS-F-T					
Camera 0	0.08	0.08	3.93	3.43	2.70
Camera 1	0.08	0.09	5.82	2.76	2.73
Mean	0.12	0.12	10.8	7.02	6.85

### IBII Tests

See Tables 13 and 14.

**Table 13:** Measurement resolution for the dynamic tests (longitudinal samples)

	u$_x$ [$\mu$m]	u$_y$ [$\mu$m]	a$_x$ x10$^6$ [m.s$^{-2}$]	a$_y$ x10$^6$ [m.s$^{-2}$]	$\varepsilon _{xx}$ [$m\varepsilon$]	$\varepsilon _{yy}$ [$m\varepsilon$]	$\varepsilon _{xy}$ [$m\varepsilon$]
1-C1-F-L	0.63	0.82	0.91	1.21	0.36	0.46	0.57
2-C1-F-L	0.83	1.07	1.20	1.54	0.48	0.59	0.74
3-C2-F-L	0.70	1.12	1.01	1.62	0.41	0.62	0.73
4-C2-F-L	0.60	1.00	0.86	1.42	0.34	0.56	0.64
5-C3-F-L	0.58	0.72	0.83	1.04	0.24	0.28	0.36
6-C3-F-L	0.59	0.74	0.84	1.06	0.24	0.30	0.39
7-C3-F-L	0.69	0.91	1.01	1.32	0.28	0.37	0.45
8-C3-F-L	0.55	0.67	0.81	0.96	0.22	0.26	0.34
9-C3-F-L	0.71	0.88	1.00	1.26	0.29	0.36	0.44
10-C4-T-L	0.77	0.95	1.11	1.34	0.47	0.55	0.69
11-C4-T-L	0.80	1.38	1.14	1.66	0.45	0.54	0.69
	0.68	0.93	0.97	1.31	0.34	0.44	0.55

**Table 14:** Measurement resolution for the dynamic tests (transverse samples)

	u$_x$ [$\mu$m]	u$_y$ [$\mu$m]	a$_x$ x10$^6$ [m.s$^{-2}$]	a$_y$ x10$^6$ [m.s$^{-2}$]	$\varepsilon _{xx}$ [$m\varepsilon$]	$\varepsilon _{yy}$ [$m\varepsilon$]	$\varepsilon _{xy}$ [$m\varepsilon$]
12-C1-F-T	0.84	1.02	1.22	1.47	0.49	0.57	0.73
13-C1-F-T	0.71	0.94	1.01	1.37	0.41	0.52	0.67
14-C1-F-T	0.75	1.02	1.09	1.46	0.45	0.57	0.70
15-C2-F-T	0.67	1.01	0.95	1.45	0.38	0.56	0.66
16-C2-F-T	0.57	1.05	0.84	1.51	0.33	0.60	0.65
17-C4-T-T	0.68	0.81	1.07	1.18	0.37	0.45	0.55
18-C4-T-T	0.64	0.76	0.93	1.14	0.36	0.42	0.56
19-C4-T-T	0.78	0.97	1.11	1.43	0.45	0.55	0.69
Mean	0.71	0.95	1.03	1.38	0.40	0.53	0.65

## Footnotes

1 https://members.loria.fr/FSur/software/gridmethod/.
